# Faculty Perceptions of Engaging Older Adults in Higher Education: The Need for Intergenerational Pedagogy

**DOI:** 10.1093/geroni/igab046.1411

**Published:** 2021-12-17

**Authors:** Jason Dauenhauer, Afeez Hazzan, Kristin Heffernan

**Affiliations:** State University of New York, Brockport, Brockport, New York, United States

## Abstract

Institutions of higher education need to become more age friendly. Creating an on-campus lifelong learning program can offer older adults opportunities to audit classes and engage in multigenerational classrooms, but can also promote intergenerational learning when instructors consciously use pedagogy that fosters engagement between learners from various generations. Promoting intergenerational learning to facilitate reciprocal sharing of expertise between generations is also the fourth principle of the Age Friendly University framework. This qualitative interview study examines the perspectives of 27 faculty members who have opened their face to face classrooms to older adult auditors to 1) Explore perceived benefits and challenges associated with having older adults in the college classroom and to 2) Determine what levels of intergenerational learning may be taking place. Compared to lecture-based courses, faculty whose pedagogy promotes discussion, sharing, and small group work reported detailed examples of older adult learners and traditionally-aged college students engaging in course-related discussion. The unique, historical and diverse perspectives of older adults improved the quality of education for students, and fostered in-depth learning. Challenges related to older adult auditors included poor/limited attendance, sharing of strong opinions/dominating class discussion, sensory/mobility and technology accessibility. Recommendations include training to promote intergenerational engagement in college classrooms.

